# Benchmarking large language models for identifying transcription factor regulatory interactions

**DOI:** 10.1093/bioinformatics/btaf653

**Published:** 2025-12-12

**Authors:** Lake Noel, Yi-Wen Hsiao, Yimeng He, Andrew Hung, Xiaojiang Cui, Edward Ray, Jason H Moore, Pei-Chen Peng, Xiuzhen Huang

**Affiliations:** Department of Computational Biomedicine, Cedars-Sinai Medical Center, Los Angeles, CA 90048, United States; Department of Computational Biomedicine, Cedars-Sinai Medical Center, Los Angeles, CA 90048, United States; Department of Computational Biomedicine, Cedars-Sinai Medical Center, Los Angeles, CA 90048, United States; Biomedical Imaging Research Institute, Cedars-Sinai Medical Center, Los Angeles, CA 90048, United States; Department of Urology, Cedars-Sinai Medical Center, Los Angeles, CA 90048, United States; Department of Surgery, Cedars-Sinai Medical Center, Los Angeles, CA 90048, United States; Department of Surgery, Cedars-Sinai Medical Center, Los Angeles, CA 90048, United States; Department of Computational Biomedicine, Cedars-Sinai Medical Center, Los Angeles, CA 90048, United States; Department of Computational Biomedicine, Cedars-Sinai Medical Center, Los Angeles, CA 90048, United States; Department of Computational Biomedicine, Cedars-Sinai Medical Center, Los Angeles, CA 90048, United States

## Abstract

**Motivation:**

Transcription factors (TFs) and their target genes form regulatory networks that control gene expression and influence diverse biological processes and disease outcomes. Although multiple computational methods and curated databases have been developed to identify TF–target interactions, they often require specialized expertise. Large language models (LLMs) chatbots offer a more accessible alternative for querying TF–target interactions. In this study, we benchmarked four prominent LLMs, Anthropic’s Claude 3.5 Sonnet, Google’s Gemini 1.0 Pro, OpenAI’s GPT-4o, and Meta’s Llama3 8b, using 8432 literature-curated human TF–target interactions. We examined four regulatory categories: bidirectional, ambiguous, self-regulated, and unidirectional interactions.

**Results:**

Under single-turn queries, Claude 3.5 Sonnet and GPT-4o outperformed the others, with balanced accuracies reaching 50.0 ± 7.6% (GPT-4o, self-regulated) and 48.2 ± 1.0% (Claude 3.5 Sonnet, unidirectional). Zero-temperature settings generally enhanced reproducibility, and multi-turn prompting improved performance for most models, increasing Claude 3.5 Sonnet’s accuracy on self-regulated pairs by 32.6%. Excluding TF–target pairs with all unknown regulation types also generally improved accuracy, with unidirectional regulation reaching near 70% balanced accuracy in some cases. We also benchmarked Anthropic’s Claude 3.5 Sonnet, Google’s Gemini 2.0 Flash, OpenAI’s GPT-4o, and Meta’s Llama3 using 5148 experimentally derived TF–target interactions. Claude 3.5 Sonnet consistently outperformed the other models across conditions. Our findings highlight that prompt engineering and strategic use of model parameters consistently influence LLM chatbots’ performance on TF–target identifications. This study establishes a benchmarking framework and demonstrates the potential of pre-trained general-purpose LLMs to support regulatory biology research, especially for researchers without extensive computational expertise.

**Availability and implementation:**

The literature-based TF–target interactions ground truth were obtained from TRRUST v2 human dataset (www.grnpedia.org/trrust). The experimental derived TF–target interactions ground truth were obtained from TFLink Home Sapiens small-scale interaction table (https://tflink.net/). Processed TF–target interactions data and the analytical pipeline has been compiled as an interactive Python notebook file and is available at https://github.com/pengpclab/LLM-TF-interactions.

## 1 Introduction

Transcription factors (TFs) are proteins that play a crucial role in regulating gene expression, particularly in controlling cell or tissue-specific gene activity, which contribute to phenotypic diversity and can drive disease onset or progression (Lambert *et al.* 2018). TFs typically bind to specific DNA sequences called transcription factor binding sites (TFBSs), activating or repressing their target genes’ expression and creating distinct spatiotemporal expression patterns (Inukai *et al.* 2017). The full set of regulatory interactions between a TF and its target genes forms a gene regulatory network, which provides a comprehensive view of transcriptional regulation on a genome-wide scale (Macneil and Walhout 2011). Disruptions of TF regulation can impair normal biological processes, potentially resulting in developmental defects or disease onsets (Weidemüller *et al.* 2021). Thus, identifying TF–target regulatory interactions not only deepens our understanding of the transcriptional mechanisms underlying complex biological processes but also sheds light on the regulation of gene expression in various cell states and diseases.

The advent of high-throughput sequencing technologies has enhanced the identification of TF–target regulations on a genome-wide scale. This progress has led to the creation of numerous databases storing these regulatory relationships, based on either experimental data or literature reviews. Experimental data-driven databases include GTRD (Kolmykov *et al.* 2021), TFtarget (Zhang *et al.* 2020) and the Harmonizome project (encompassing ENCODE, TRANSFAC, JASPAR, and ChEA) (Diamant *et al.* 2025), while literature-based databases comprise TRRUST (Han *et al.* 2018), TFLink (Liska *et al.* 2022), and hTFtarget (Zhang *et al.* 2020). As data accumulation accelerates, computational methods such as tREMAP (Lim and Xie 2018), InteGRaNet (Chang *et al.* 2013), and TGPred (Cao *et al.* 2023) have emerged to expedite TF–target identifications. The rising popularity of artificial intelligence (AI) chatbots presents a promising new opportunity for querying TF–target regulatory interactions without the need to manually search through individual databases. These tools may offer a more user-friendly solution, lowering the barrier for researchers who may not have extensive experience with computational modeling or data acquisition.

AI chatbots powered by large language models (LLMs) have transformed academic research by automating literature reviews, enhancing data analysis, and fostering interdisciplinary research (Meyer *et al.* 2023). LLMs are neural network models built upon vast repositories of human knowledge, providing a foundation for numerous downstream applications and making modeling processes more feasible, efficient, and scalable. Inspired by the capabilities of LLMs, researchers have begun leveraging pre-trained AI chatbots for a range of biomedical applications, including clinical decision support (Liu *et al.* 2023, Lee *et al.* 2025), biomedical education (Kung *et al.* 2023, Piccolo *et al.* 2023), the generation of simplified radiology reports for patient comprehension (Jeblick *et al.* 2022), and addressing questions in human genetics. These examples demonstrate the immense potential of LLMs in advancing biomedical research.

In this study, we investigate the potential of general-purpose (non-fine-tuned) LLMs to identify TF–target regulatory interactions or the regulation types. Our focus is on assessing their baseline performance, suitability in resource-limited settings, and accessibility for non-expert users. We evaluate four prominent LLMs, including Anthropic’s Claude 3.5 Sonnet (Caruccio *et al.* 2024), Google’s Gemini 1.0 Pro (Imran and Almusharraf 2024), OpenAI’s GPT-4o (OpenAI 2024), and Meta’s Llama3 (Touvron *et al.* 2023). This benchmarking is based on 8432 pairs of literature curated human TF–target regulatory interactions from the Transcriptional Regulatory Relationships Unraveled by Sentence-based Text-mining (TRRUST) v2 database (Han *et al.* 2018), including bidirectional, unidirectional, self-regulated, and ambiguous regulation directions as well as activation, repression and unknown regulation types. We also assess the consistency of responses and implement multi-turn prompting techniques. To address potential data leakage from literature curated TF–target interactions, we evaluate LLMs performance on 5148 pairs of experimentally derived TF–target interactions, which show comparable results. For this, we use the same four models, replacing Gemini 1.0 Pro with Gemini 2.0 Flash due to model availability at the time of testing.

## 2 Materials and methods

### 2.1 Regulatory interactions data from literatures and experiments

**Figure 1.**
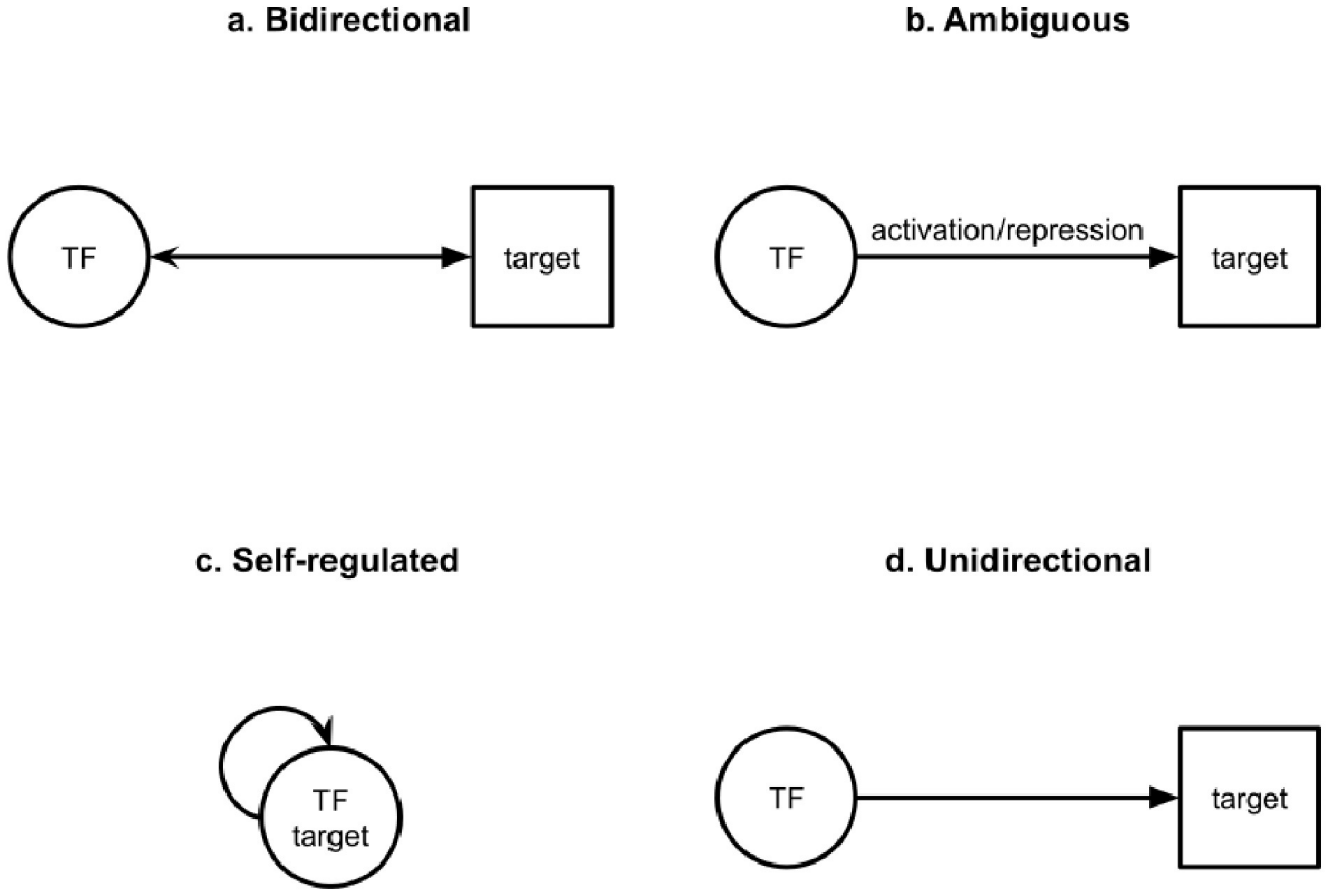
Schematic examples of TF–target interaction categories. (a) Bidirectional: pairs listed as TF-to-target and then reversed, i.e. the target is the TF and vice versa; (b) ambiguous: pairs listed in only one direction and with multiple regulation types; (c) self-regulated: pairs in which the TF regulates itself; (d) unidirectional: all remaining pairs, those listed as TF-to-target in one direction with a single regulation type.

**Figure 2.**
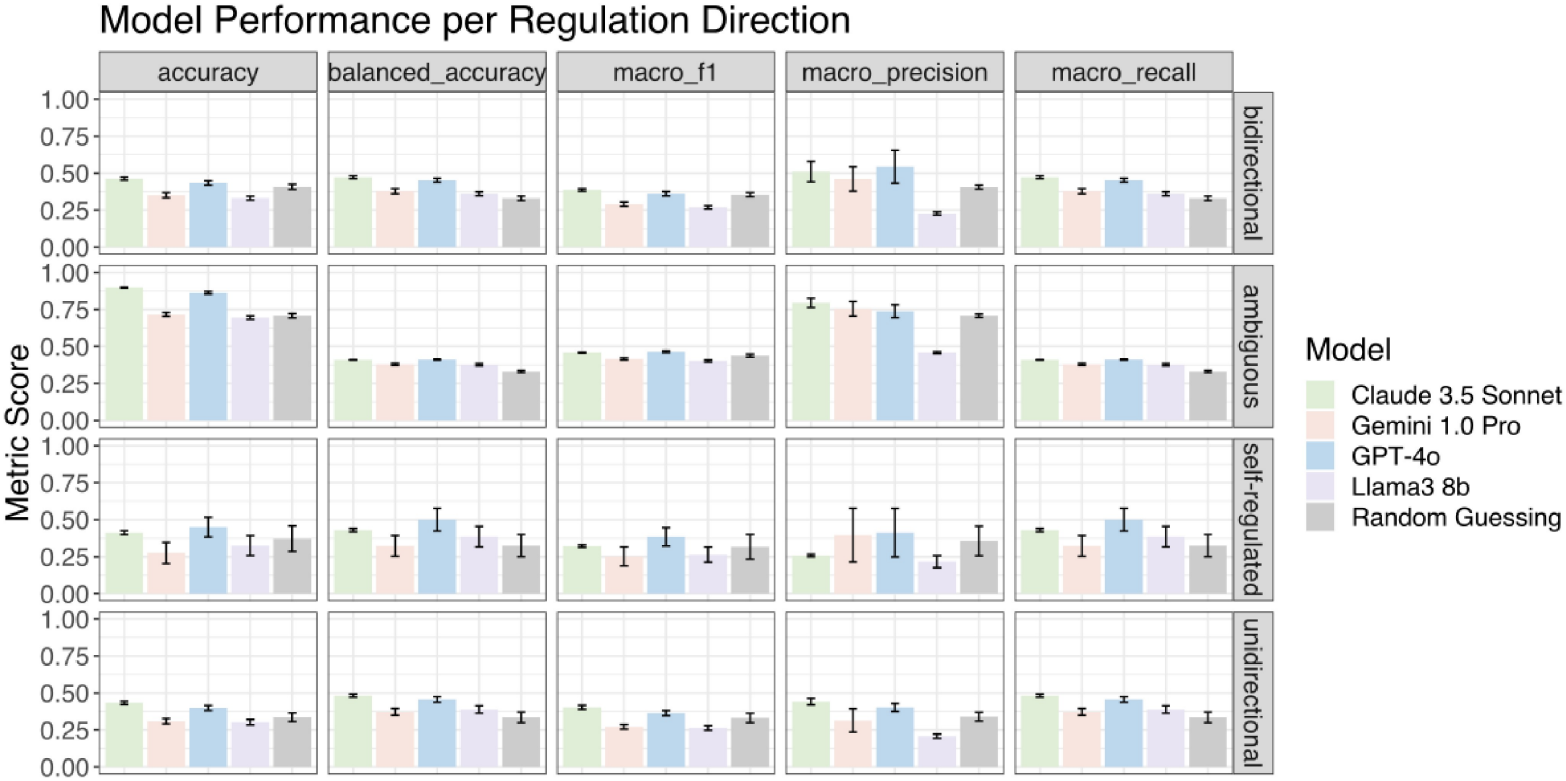
Models’ performance and standard deviation by regulation direction. Accuracy, balanced accuracy, macro F1, macro precision, and macro recall are reported for the four LLMs (Claude 3.5 Sonnet, Gemini 1.0 Pro, GPT-4o, and Llama3 8b). The LLM performances are also compared to simulated random guessing baseline (Random Guessing). Performance is shown separately for each regulation direction category: bidirectional, ambiguous, self-regulated, and unidirectional. Error bars indicate standard deviations across 10 iterations.

We used the human TF–target regulatory interactions data from TRRUST v2 (7) and TFLink (Han *et al.* 2018). TRRUST v2 is a manually curated, literature-based database, making it an ideal benchmarking dataset. For the TRRUST v2 human dataset, each row consists of the transcription factor, the target, the regulation type (either activation, repression, or unknown), and the PubMed IDs of articles that document these interactions. Within the TRRUST dataset, we defined four regulation directions: bidirectional (206 pairs), ambiguous (801 pairs), self-regulated (24 pairs), and unidirectional (7390 pairs) (Fig. 1). A detailed breakdown of the regulation types of each category is shown in Fig. 1, available as supplementary data at *Bioinformatics* online. For unidirectional pairs, we chose a single 250-pair subset mainly to reduce computational resources and complexity and secondarily to roughly match the number of pairs of the other direction types. To show how well our single set of 250 samples represents the entire dataset of 7390 TF–target pairs, we also randomly sampled 10 sets of 250 pairs and had the model iterate over them one time, as opposed to 10 times for the other subsets and regulation directions. The similar performance across sampling schemes indicates that a single set of 250 TF–target pairs is a reliable representative of the full dataset of 7390 unidirectional TF–target pairs (Fig. 2, available as supplementary data at *Bioinformatics* online).

To address the potential issue of data leakage of LLM chatbots, we used the small-scale human dataset from TFLink (Han *et al.* 2018) to curate the experimentally derived TF–target interactions. TFLink combines TF–target interactions from multiple databases and provides information on the sources of the database, experimental methods, and publications. There is no TF–target interaction in the TFLink small human dataset reported only by experimental evidence but not literature. We identified 5148 TF–target pairs reported by GTRD (Kolmykov *et al.* 2021) (interactions derived from ChIP-seq binding data), or DoRoTHea (Garcia-Alonso *et al.* 2019, Badia-I-Mompel *et al.* 2022, Müller-Dott *et al.* 2023) (interactions derived from ChIP-seq and bulk RNA-seq data), but not TRRUST. For comparison, we also identified 2973 TF–target pairs reported by TRRUST only. For the TFLink dataset, each row is a confirmation of an interaction consisting of a transcription factor and its target, but lacking information on regulation type.

### 2.2 Pre-trained large language models

We used four primary LLMs: Anthropic’s Claude 3.5 Sonnet (17) (2024-06-20 version via API), Google’s Gemini 1.0 Pro (18) (gemini-1.0-pro-latest version via API, last accessed 2024-07-24), Open AI’s GPT-4o (19) (2024-05-13 version via API), and Meta’s Llama3 8b (20) [llama3:8b via the Ollama framework (21)]. For Llama3, we used the 8b model through Ollama, a framework that enables LLMs to be run on one’s local hardware. Due to hardware constraints and to better reflect resource limited environments typical for personal computers setups, we used the 8b model rather than the larger 70b version. The other models were run on their respective servers (Claude 3.5 Sonnet on Anthropic’s servers, Gemini 1.0 Pro on Google’s servers, and GPT-4o on OpenAI’s servers). All the analyses in this study were conducted on a machine with an Apple M2 chip, 16GB of memory, and no dedicated GPU. Like Open AI’s GPT-4o, Claude 3.5 Sonnet is considered both the most intelligent and most cost effective among Anthropic’s offered models. For TFLink data, we used Gemini 2.0 Flash (gemini-2.0-flash-001 version via API, last accessed 2025-06-26) in place of Gemini 1.0 Pro due to availability.

### 2.3 Model parameters and system prompt settings

To test the parameters for reproducible research, we investigated the temperature parameter and seed. The temperature parameter controls the randomness of the model’s output; a value of zero forces the model to be fully deterministic, always producing the highest-probability answer. Unless otherwise specified, all models were tested at their default temperature settings. For all models, we did not set a seed, which theoretically could be used for more deterministic and therefore more reproducible results. Google’s Gemini 1.0 Pro does not have a seed option. Open AI, while it features a seed parameter, does not guarantee determinism with the use of this parameter (22). Indeed, with GPT-4o, setting a seed and setting the temperature to zero still resulted in deviation in accuracy.

We also used system prompts to guide the LLMs overall behavior and role. Gemini 1.0 Pro does not have the option to set a system prompt, and attempting to simulate the option via multi-turn chat resulted in lower accuracy. For those models (GPT-4o, Claude 3.5 Sonnet, and Llama3 8b) that had the option, we initialized the API requests with the following system prompt: “You are a molecular biologist’s helpful assistant who uses documented research.” We chose this system prompt because, particularly for Llama3 8b, this system prompt resulted in highest accuracy compared to not defining a prompt and “You are a molecular biologist’s helpful assistant.”

### 2.4 User prompts for each testing scenario

The final user prompts we used in each testing scenario are described below. For the benchmarking, reproducibility, and the unknown regulation type exclusion tasks, the user prompt was:

*How does transcription factor {TF} regulate {target} in humans? Restrict your answer to one word and nothing more: repression, activation, or unknown.*

For the multi-turn task, the user prompts were:

1. Explain how {TF} regulates {target} in humans.
2. Based on your previous reasoning, is the interaction between {TF} and {target} activation, repression, or unknown?
3. Restrict your answer to one word: repression, activation, or unknown.

For the experimental-derived TF–target interactions task, the user prompt was:

*Does transcription factor {TF} regulate {target} in humans? Restrict your answer to one word and nothing more: yes or no/unknown.*

The restriction on the query was intended to constrain the model’s response to our desired format, either a single regulation type or a simple yes/no answer. Without this constraint, the models tended to generate paragraph-length responses with detailed reasoning.

We also tested the effect of including the word “please” in user prompts for Llama3 8b, as prior work has shown that such phrasing can influence its responses (Yin *et al.* 2024). Specifically, we compared two user prompts:

With “Please”: *How does transcription factor {tf} regulate {target} in humans? Please restrict your answer to one word and nothing more: repression, activation, or unknown.*

Without “Please”: *How does transcription factor {tf} regulate {target} in humans? Restrict your answer to one word and nothing more: repression, activation, or unknown.*

### 2.5 Evaluation criteria and metrics

For the literature-based TRRUST TF–target interactions, the model was instructed to respond with only one regulation type: activation, repression, or unknown. We considered the model’s response correct if the response matched one of the ground truth regulation types. For experimentally derived TF–target interactions, we consider the model’s response correct if the response was aligned with the ground truth regulation direction: yes or no.

The evaluation metrics used in this study include accuracy, balanced accuracy, macro F1, macro precision, and macro recall. Accuracy measures the overall proportion of correct predictions. Balanced accuracy accounts for class imbalance by averaging the recall across all classes. Macro precision, macro recall, and macro F1 treat each class equally by computing the metric independently for each class and then averaging the results, regardless of class frequency. This approach ensures that performance is not dominated by the majority class, making it especially appropriate for imbalanced datasets.

## 3 Results

### 3.1 Benchmarking LLMs performance on TF–target interactions

We assessed the performance of four pre-trained LLMs by benchmarking them using manually curated TF–target regulatory interactions from the TRRUST v2 database (7), a comprehensive and widely recognized gold standard for human TF–target interactions. Within the TRRUST dataset, we identified four regulation directions: bidirectional (206 pairs), ambiguous (801 pairs), self-regulated (24 pairs), and unidirectional (7399 pairs from which we randomly selected 250 pairs) (Fig. 1). These categories were distinguished by their regulation direction and regulation types. Bidirectional regulation occurs when a transcription factor regulates a target, and the target, in turn, regulates the TF. Ambiguous pairs involve TFs regulating a target, but the regulation types could be a combination of activation, repression, or unknown from different sources of verifiable literature evidence. Here, “unknown” denotes an interaction of unspecified or context-dependent effect, rather than the absence of any interaction. Self-regulated pairs occur when a TF regulates itself. Finally, unidirectional regulation, the most common type, occurs when a TF regulates a target in one direction with a single regulation type, which may be activation, repression, or unknown.

To evaluate the information retrieval ability of the four LLMs, Claude 3.5 Sonnet, Gemini 1.0 Pro, GPT-4o, and Llama3 8b, we used the following query:

*How does transcription factor {TF} regulate {target} in humans? Restrict your answer to one word and nothing more: repression, activation, or unknown.*

As such, while there can be multiple ground truth regulation types, the model is asked to respond with only one answer. We consider the model’s response correct if the response is one of the ground truth regulation types.

**Figure 3.**
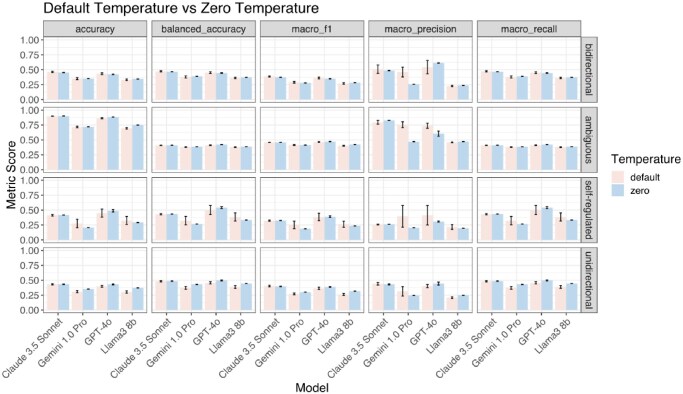
Performance at default temperature versus zero temperature. Each panel shows a different evaluation metric (accuracy, balanced accuracy, macro F1, macro precision, and macro recall) for four language models (Claude 3.5 Sonnet, Gemini 1.0 Pro, GPT-4o, and Llama3 8b) under two temperature settings: default (pink) and zero (blue). Performance is stratified by regulation direction category: bidirectional, ambiguous, self-regulated, and unidirectional. Error bars represent standard deviations across 10 iterations.

For each regulation direction, we queried the model 10 times on the same TF–target pairs. Figure 2 presents the performance and standard deviation for each model across five evaluation metrics: accuracy, balanced accuracy, macro F1 score, macro precision, and macro recall. Overall, Claude 3.5 Sonnet had the best balanced accuracy among the four models, except in the case of self-regulated pairs, where GPT-4o performed slightly better. Across all regulation direction categories, each model outperformed random guessing, with average balanced accuracies of 44.8 ± 3.5% for Claude 3.5 Sonnet, 36.3 ± 2.7% for Gemini 1.0 Pro, 45.5 ± 3.7% for GPT-4o, and 37.8 ± 1.2% for Llama3 8b, compared to 33.0 ± 0.4% for random guessing. Also, standard deviations were consistent across all models, except for the self-regulated groups. It is also worth noting that Claude 3.5 Sonnet exhibited a low standard deviation for each regulation direction group and outperformed other models in providing concise responses. Unlike the other models, which occasionally returned additional words or full sentences, Claude 3.5 Sonnet consistently adhered to the one-word format (“Activation,” “Repression,” or “Unknown”) 100% of the time. A reason for Llama3 8b’s relatively low macro precision could be due to the model’s propensity for not responding with unknown, as shown in the performance breakdown by regulation type in Fig. 3, available as supplementary data at *Bioinformatics* online.

As a control, we simulated random guessing by randomly assigning each query with one of the three possible responses: “Activation,” “Repression,” or “Unknown,” repeating this test for 10 iterations. The mean accuracy and standard deviation from random guessing for each regulatory direction category were as follows: bidirectional 40.7% ± 1.7%, ambiguous 70.8% ± 1.5%, self-regulated 37.1% ± 8.7%, unidirectional 33.6% ± 2.9% (Fig. 2). Claude 3.5 Sonnet and GPT-4o consistently outperformed random guessing across most regulation categories, indicating their ability to capture biologically relevant TF–target interactions.

We recognized crafting effective prompts was the key to guide LLMs to generate the desired responses. Therefore, we tested multiple prompt variations before finalizing the prompt used in this study. With our earlier version of the query, “How does transcription factor {TF} regulate {target} in humans? Please restrict your answer to one word: repression, activation, or unknown.”, Llama3 8b would often answer in sentences or paragraphs, sometimes including references, rather than the single word we were looking for. To enforce brevity, we tested three approaches. First, setting a lower “max_tokens” output to restrict the number of characters resulted in more concise answers but lower accuracy. Similarly, adding “You answer with only one word” to the system prompt also resulted in more concise answers but lower accuracy. In contrast, adding “and nothing more” at the end of the query improved both accuracy and concise response.

Even with this finalized prompt, models still occasionally answered with something other than “repression,” “activation,” or “unknown” (e.g. “induction,” “autoactivation,” “suppression,” “activates,” or, more rarely, a sentence), but these instances were less than 2% of the model’s responses for the given regulation direction. Interestingly, in tests with Llama3 8b, removing “Please” from the query resulted in better accuracy (Table 1, available as supplementary data at *Bioinformatics* online).

### 3.2 Setting zero temperature parameter for reproducible research

LLMs have various parameters that can be set, including temperature. Temperature adjusts the variability of the models’ responses, zero temperature being the least variable and therefore more conducive to reproducible results. Here, we repeated the TF–target interactions tests, setting the temperature to zero.

Consistent with the default temperature results, all models performed similarly across four regulation direction types in balanced accuracy, with Claude 3.5 Sonnet and GPT-4o leading in almost all cases. Though the zero temperature balanced accuracies were slightly lower than those at default temperature for some regulation types, such as bidirectional pairs for Claude 3.5 Sonnet (47.2 ± 1.0% default, 46.6 ± 0.2% zero) and GPT-4o (45.2 ± 1.3% default, 44.5 ± 0.5% zero), and self-regulated pairs for Gemini 1.0 Pro (32.3 ± 7.0% default, 26.7 ± 0% zero) and Llama3 8b (38.5 ± 6.9% default, 33.3 ± 0% zero), the performance between default and zero temperature was broadly similar or even slightly improved in some cases (Fig. 3). For example, all models showed better balanced accuracies on ambiguous pairs, Gemini 1.0 Pro and Llama3 8b performed better on bidirectional pairs, GPT-4o excelled with self-regulated pairs, and all models showed improvement on unidirectional pairs. For self-regulated TF–target pairs, Gemini 1.0 Pro showed a noticeable decrease (∼5.6%) in balanced accuracy between default temperature and zero temperature. Also, both Llama3 8b and Gemini 1.0 Pro showed a standard deviation of zero across all regulation directions at zero temperature.

### 3.3 Enhancing performance with multi-turn prompting

The LLM APIs are stateless; they cannot remember previous queries and responses. We simulated the state by sending the conversation history with each query. This allows the model to make use of information it gathers from prior queries, emulating the turn-based conversation of a chatbot. Since this method leads the model to articulate and use its own reasoning, we hypothesized this would result in higher accuracy than our single-turn prompt.

Since there are so few self-regulated TF–target pairs and multi-turn prompting requires multiple queries, meaning more processing required than a single-turn prompt, we found it more expedient to test the models on self-regulated pairs only. For each model, we used the following queries per multi-turn prompt:

1. Explain how {TF} regulates {target} in humans.
2. Based on your previous reasoning, is the interaction between {TF} and {target} activation, repression, or unknown?
3. Restrict your answer to one word: repression, activation, or unknown.

The first query prompts the model to reason about the TF–target regulation. The second query asks the model to infer the regulation type, taking into account the model’s reasoning from the first query. The final query is meant to make the model answer within our desired format of a single regulation type only.

**Figure 4.**
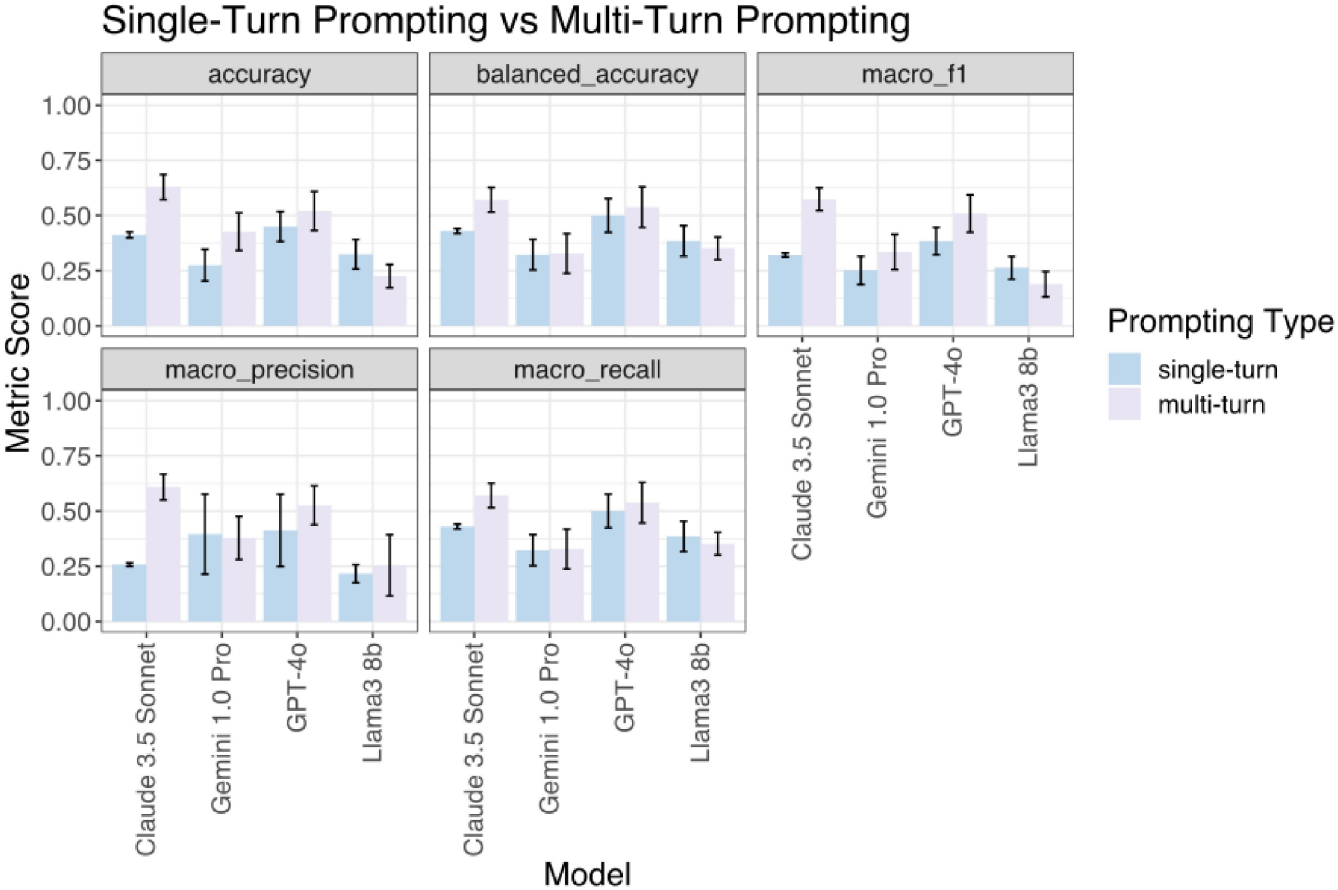
Performance comparison of single-turn prompting versus multi-turn prompting. Performance metrics (accuracy, balanced accuracy, macro F1, macro precision, and macro recall) are shown for four language models (Claude 3.5 Sonnet, Gemini 1.0 Pro, GPT-4o, and Llama3 8b) under two prompting strategies: single-turn (blue) and multi-turn (purple). Bars represent mean scores across 10 iterations, and error bars indicate standard deviation. Multi-turn prompting generally improves performance, particularly for Claude 3.5 Sonnet and Gemini 1.0 Pro, highlighting the impact of conversational context on model accuracy and consistency.

We conducted this analysis for 10 iterations and compared the performance of single-turn prompting versus multi-turn prompting (Fig. 4). Multi-turn prompting led to higher accuracy and balanced accuracy across all models, except for Llama3 8b, whose accuracy decreased. This approach was especially effective for Claude 3.5 Sonnet, yielding a 50% increase in accuracy and 30% increase in balanced accuracy. However, multi-turn prompting also introduced greater variability across iterations, as reflected by a higher standard deviation compared to single-turn prompting.

To examine the influence of the temperature parameter on multi-turn prompting, we repeated the tests with the temperature set to zero. Similar to results observed at default temperature, multi-turn prompting improved accuracy across all models except Llama3 8b (Fig. 4, available as supplementary data at *Bioinformatics* online). The magnitude of improvement remained consistent between zero and default temperature settings. We observed larger standard deviations in performance compared to single-turn prompting. The results suggest that while multi-turn prompting enhanced accuracy, it may also introduce greater response instability.

### 3.4 Excluding unknown regulation types improves model accuracy

Accurate identifications of TF–target regulatory interactions rely on two components: the interaction directions and the interaction types. Interaction types can be categorized as activation, repression, or unknown. The “unknown” category can arise from various factors, such as a lack of validation in biological experiments, dual regulatory roles, or tissue-specific changes. To determine whether accuracy would improve with the exclusion of TF–target pairs of unknown regulation type, we removed such pairs after querying the models. We tested two exclusion settings:

1. Broad Unknown Exclusion: excluding TF–target pairs for which the ground truth regulation type includes “unknown” in combination with another category. For examples, “activation; unknown” and “repression; unknown.”
2. Strict Unknown Exclusion: excluding TF–target pairs for which the ground truth regulation type in TRRUST is strictly “unknown”.

**Figure 5.**
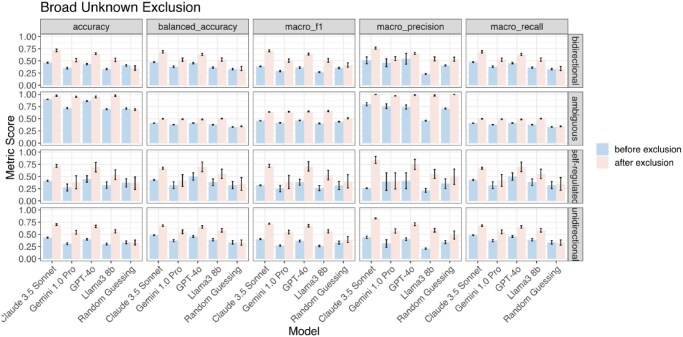
Performance of retaining all cases of ground truth unknown regulation types (before exclusion) versus excluding them (after exclusion). Performance is evaluated using five metrics, accuracy, balanced accuracy, macro F1, macro precision, and macro recall, across four regulatory categories: bidirectional, ambiguous, self-regulated, and unidirectional. Each panel compares model performance before (blue) and after (pink) excluding TF–target pairs under setting 1: Broad Unknown Exclusion. Models include Claude 3.5 Sonnet, Gemini 1.0 Pro, GPT-4o, and Llama3 8b, and a random guessing baseline. The standard deviations across 10 iterations are also reported.

The results demonstrate that Broad Unknown Exclusion (Fig. 5) improves model balanced accuracy across all regulation directions. The most substantial increases were observed in self-regulated, unidirectional, and bidirectional interactions, where balanced accuracy improvements ranged from approximately 9.5%–24.1%. Specifically, GPT-4o and Claude 3.5 Sonnet showed the highest overall performance, achieving over 60% balanced accuracy in self-regulated and bidirectional categories when all cases of unknown regulation types were removed. On the other hand, the Strict Unknown Exclusion mainly improves the balanced accuracy in the unidirectional category (Fig. 5, available as supplementary data at *Bioinformatics* online).

**Figure 6.**
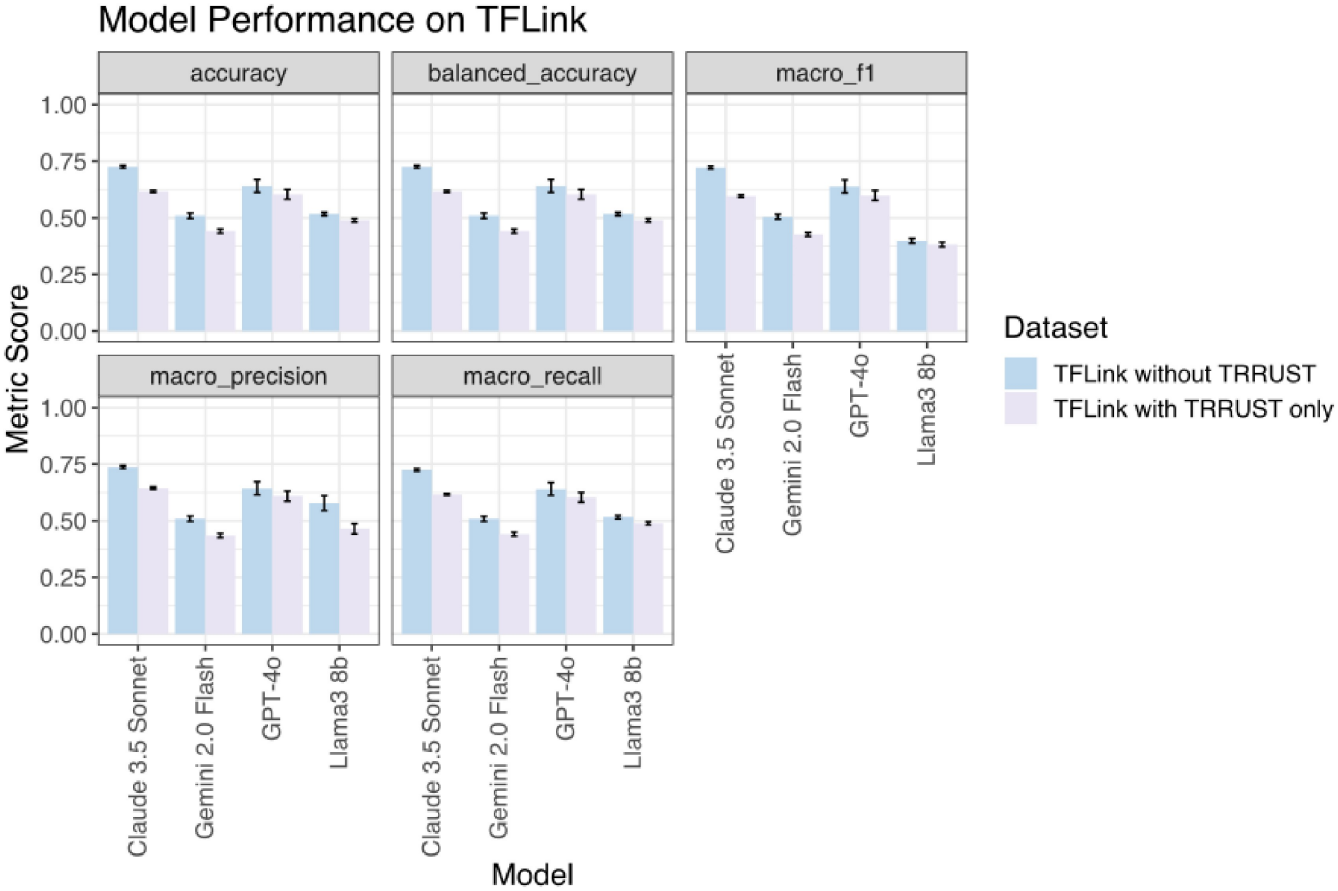
Performance on experimentally derived TF–target interactions from TFLink, comparing datasets with and without TRRUST. Performance metrics (accuracy, balanced accuracy, macro F1, macro precision, and macro recall) are shown for four language models (Claude 3.5 Sonnet, Gemini 2.0 Flash, GPT-4o, and Llama3 8b) for two datasets: TFLink without TRRUST (blue) and TFLink with TRRUST only (purple). Bars represent mean scores across 10 iterations and error bars indicate standard deviation. Particularly for Claude 3.5 Sonnet and Gemini 2.0 Flash, models performed better on TFLink without TRRUST dataset. Given a balanced dataset and binary classification, baseline accuracy is 50%, and balanced accuracy is equivalent to accuracy.

Setting the temperature parameter to zero successfully reduced variability across iterations, as reflected by the lower standard deviations in balanced accuracy across all models, in both Broad Unknown Exclusion (Fig. 6, available as supplementary data at *Bioinformatics* online) and Strict Unknown Exclusion (Fig. 7, available as supplementary data at *Bioinformatics* online) settings. Llama3 8b and Gemini 1.0 Pro exhibited no variation across iterations. While GPT-4o and Claude 3.5 Sonnet still showed slight variation in some categories, their standard deviations were considerably lower than at default temperature. Balanced accuracy trends remained consistent with the default temperature setting. Overall, these findings highlight the importance of filtering uncertain regulatory interactions to enhance LLM-based TF–target identifications accuracy, with Claude 3.5 Sonnet and GPT-4o consistently outperforming other models across all conditions except Broad Unknown Exclusion for ambiguous TF–target pairs, for which Llama3 8b performed the best.

### 3.5 Evaluating LLMs’ performance on experimentally derived TF–target pairs

To assess the ability of LLMs to query and identify TF–target interactions from experimentally derived data, we evaluated model performance using the small-scale human dataset from TFLink (Liska *et al.* 2022). TFLink combines TF–target interactions from multiple databases, sourced from literature-based evidence and experimentally verified TF–target pairs. This dataset does not annotate regulatory type (activation, repression, or unknown); instead, the presence of a TF–target pair denotes a known regulatory interaction. As such, we framed this task as a binary classification problem. We constructed two separate positive sets:

1. TFLink without TRRUST: 5148 TF–target interactions supported by experiment sources including GTRD (Kolmykov *et al.* 2021) and DoRoTHea (Garcia-Alonso *et al.* 2019, Badia-I-Mompel *et al.* 2022, Müller-Dott *et al.* 2023), but not TRRUST.
2. TFLink with TRRUST only: 2973 TF–target interactions sup-ported only by TRRUST.

For both positive datasets, we followed a similar approach to the unidirectional TF–target pairs. We randomly sampled one set of 250 pairs and had the models iterate over them 10 times. Then, to generate a negative class, we synthesized non-interacting TF–target pairs by independently shuffling TFs and targets, removing any pairs found in either the TFLink small- or large-scale human datasets, removing any possible resulting duplicates, then randomly selecting 250 pairs from the resulting set of 5630. To ensure consistent comparison across model evaluations, this same 250 TF–target negative set was paired with both positive datasets, from which we also randomly sampled 250 TF–target pairs.

Given the possibility that some synthetic negative pairs may represent true but unannotated interactions, we allowed for a third response option: “unknown.” The user prompt used was:

*Does transcription factor {TF} regulate {target} in humans? Restrict your answer to one word and nothing more: yes or no/unknown.*

Although the prompt accepted three responses, we treated this as a binary classification task during evaluation by grouping “no” and “unknown” into a single negative class. After querying the models on each class (negative and positive) individually, for each model and dataset (TFLink without TRRUST and TFLink with TRRUST only), we combined the responses of each class and evaluated the results.

The LLMs performed well on the experimental derived TF–target interactions testing (Fig. 6). Claude 3.5 Sonnet achieved the highest balanced accuracy: 72.6 ± 0.6% without TRRUST, and 61.6 ± 0.4% TRRUST only. This was followed by GPT-4o 64.0 ± 2.8% without TRRUST and 60.4 ± 2.2% TRRUST only. Gemini 2.0 Flash (50.9 ± 11.1% without TRRUST, 44.1 ± 0.9% TRRUST only) was on par with Llama3 8b (51.7 ± 0.7% without TRRUST and 48.9 ± 0.8% TRRUST only). Both were at or below the performance of random guessing, which would be expected to be 50% given a balanced dataset and binary classification. Across both positive datasets, Claude 3.5 Sonnet consistently outperformed the other models, with highest scores in all evaluated metrics with the exception of macro F1 for TRRUST only, for which GPT-4o outperformed. Performance was generally better on the TFLink dataset without TRRUST compared to the TRRUST-only dataset. This suggests that interactions curated from sources like GTRD and DoRoTHea may be more consistent with the language models’ learned representations or training distribution than those from literature-based TRRUST TF–target interactions. Llama3 8b exhibited the lowest performance overall, particularly in macro F1 and recall, indicating difficulty in correctly identifying true interactions.

To show how well our single set of 250 samples represents the entire dataset, for both positive and negative sets, we randomly sampled 10 sets of 250 pairs for the models to iterate over once. We found that the performance metrics across these sets showed minimal variation, with low standard deviations, indicating that our results are robust to the specific random sampling approach (Fig. 8, available as supplementary data at *Bioinformatics* online).

## 4 Discussion

In this study, we investigated how effectively pre-trained large language models (LLMs) can identify TF regulatory interactions, a critical element in gene regulatory networks. Using the manually curated TRRUST v2 database, we evaluated Claude 3.5 Sonnet, Gemini 1.0 Pro, GPT-4o, and Llama3 8b across bidirectional, ambiguous, self-regulated, and unidirectional regulatory categories. We established baseline results by assessing model performance under default settings, followed by testing at zero temperature to enhance reproducibility. Our findings demonstrate that multi-turn prompting enhances TF–target identifications for self-regulated regulatory interactions, likely because the models can incorporate prior context and reasoning across turns. Furthermore, excluding TF–target pairs with unknown regulatory roles further bolstered accuracy. For experimentally derived TF–target interactions data, we found Claude 3.5 Sonnet consistently outperformed the other models across conditions.

Unlike domain-specific models, which require fine-tuning on biomedical datasets, our study exclusively used general-purpose pre-trained LLMs. This approach reduces the need for domain-specific expertise, making these tools accessible to a broader audience. While domain-specific models can incorporate specialized knowledge, they risk overfitting and may perform suboptimally on tasks. Our results demonstrate that pre-trained LLMs with well-engineered prompts and thoughtful parameter optimization can achieve reasonable TF–target identification accuracy without extensive customization.

Prompt engineering emerged as a critical factor in guiding LLMs toward accurate predictions. The nuanced phrasing and specificity of prompts strongly influenced outcomes for ambiguous TF–target interactions. Ambiguous pairs presented the greatest challenge due to their mixed regulatory signatures, yet they also demonstrated the highest accuracy across all models, suggesting that LLMs are adept at capturing contextual complexity when properly guided. Moreover, multi-turn prompting to simulate reasoning steps further enhanced model accuracy, particularly for challenging categories such as self-regulated interactions. However, this approach introduced greater variability in predictions. These findings underscore the importance of carefully optimizing LLM interactions to balance accuracy and consistency.

While LLMs offer a scalable and user-friendly approach to TF–target interaction identifications, several limitations remain. The models occasionally produced hallucinations and biologically inconsistent responses, reflecting their reliance on pre-trained knowledge bases. Also, there is a potential risk of data leakage, as TRRUST is a literature-based resource and LLMs may have been trained on overlapping texts. While the extent of this overlap is unclear due to limited transparency in LLM training data. Current benchmarks lack high-quality, cell type-specific TF–target interaction data, limiting our ability to assess model performance in a cell type-specific manner. These limitations highlight the continued need for biological experiments to establish ground truth and validate computational predictions.

We view the benchmarking of regulatory interactions as a hierarchy of increasingly difficult questions: (i) whether a TF regulates a given gene, (ii) the type of regulation for a known TF–target pair, and (iii) identifying the downstream targets of a given TF. This study focuses on questions (i) and (ii) due to the availability of reliable, curated ground truth data. Future work should aim to expand LLM applications to questions (iii), which will require more comprehensive ground truth datasets and carefully designed evaluation frameworks. Addressing this challenge will likely involve the integration of single-cell or spatial omics data. Such tasks will require integrating LLM predictions with high-dimensional datasets through advanced matrix processing and multimodal data fusion techniques. Moreover, developing explainable AI frameworks will be essential for improving the interpretability of LLM predictions, enabling their integration into precision medicine and other translational research applications.

## Supplementary Material

btaf653_Supplementary_Data
