# Supplementary material for: Benchmarking large language models for identifying transcription factor regulatory interactions
Source: Bioinformatics. 2025 Dec 12;42(1):btaf653. doi: 10.1093/bioinformatics/btaf653 (PMC12766914; doi:10.1093/bioinformatics/btaf653)
Supplement: btaf653_Supplementary_Data [file btaf653_supplementary_data.docx]

**Supplementary Information: Benchmarking Large Language Models for Identifying Transcription Factor Regulatory Interactions**

| 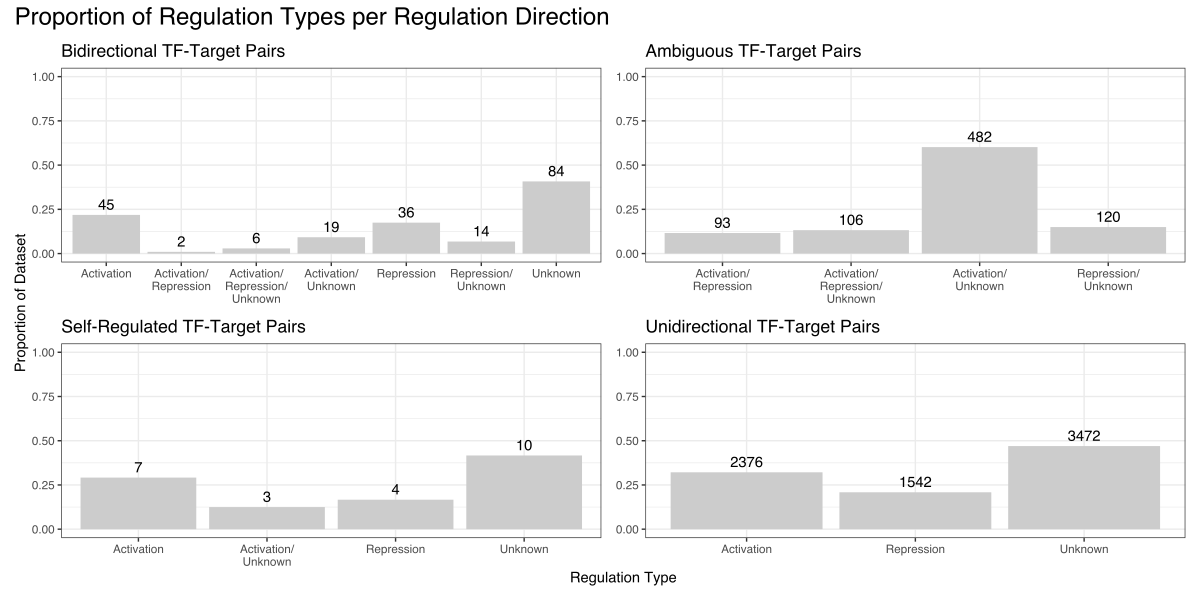 |
| --- |
| Supplementary Figure 1. The proportion and number of regulation types per regulation direction in TRRUST v2 human dataset. |
| 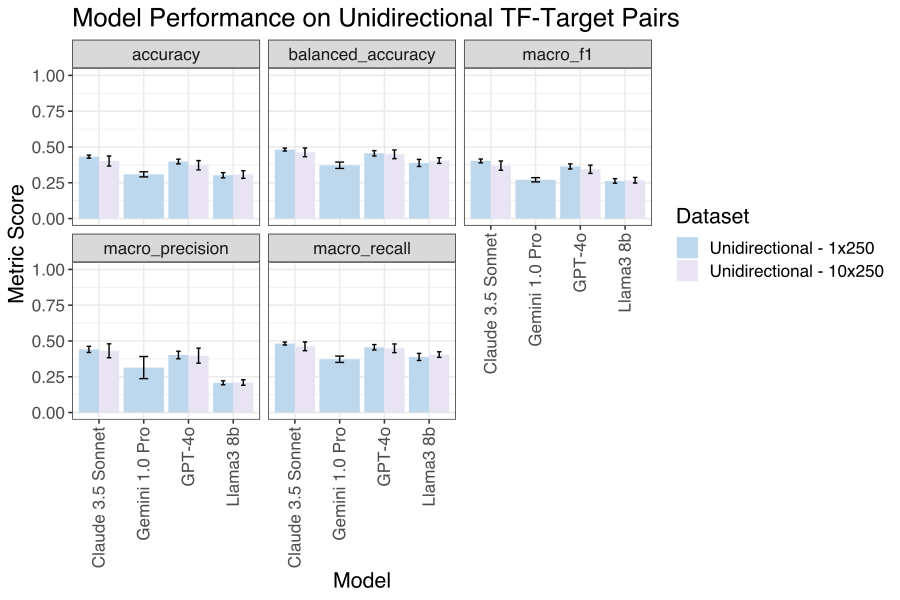 |
| **Supplementary Figure 2. Model performance on unidirectional TF-target pairs across different sampling strategies.** Bar plots show five performance metrics (accuracy, balanced accuracy, macro F1, macro precision, and macro recall) for three large language models (Claude, GPT, and Llama), evaluated using two sampling schemes: a single set of 250 unidirectional TF-target pairs evaluated over 10 iterations (blue), and 10 independently sampled sets of 250 pairs each evaluated once (purple). Error bars represent standard deviations across iterations. The similar performance across sampling schemes indicates that a single set of 250 TF-target pairs is a reliable representative of the full dataset of 7,390 unidirectional TF-target pairs. Gemini 1.0 Pro was unavailable at the time the 10 independent sets were evaluated. |
| 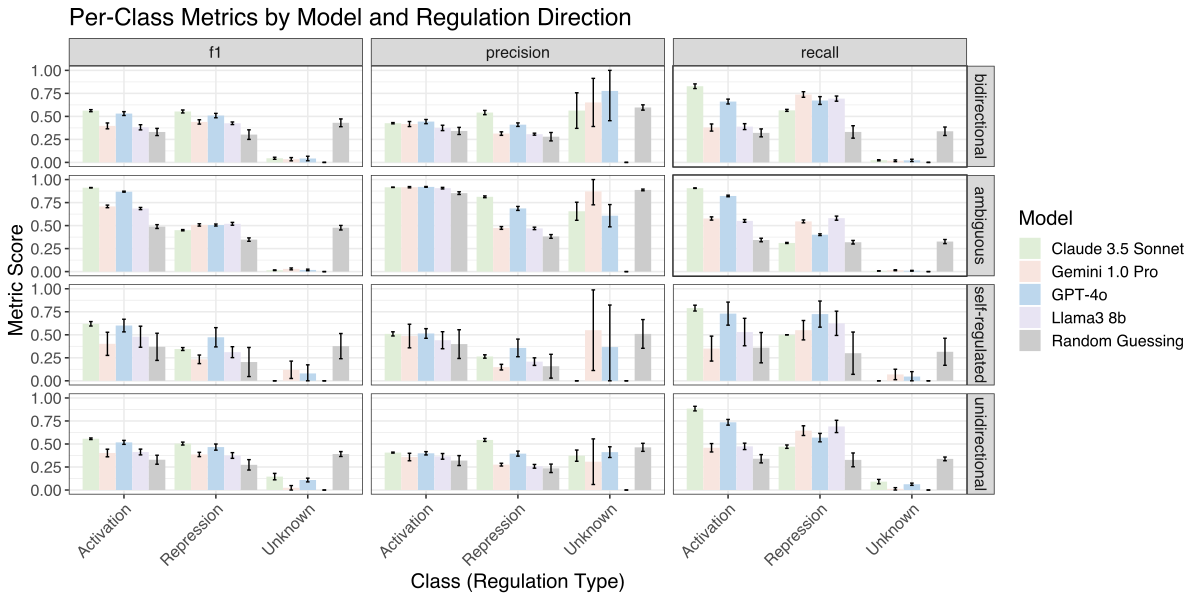 |
| **Supplementary Figure 3. Models’ performance and standard deviation by regulation type.** F1, precision, and recall are reported for the four LLMs (Claude, Gemini, GPT-4o, and Llama), separated by the activation, repression, and unknown regulation types. The LLM performances are also compared to simulated random guessing baseline (Random Guessing). Performance is shown separately for each regulation direction category: bidirectional, ambiguous, self-regulated, and unidirectional. Error bars indicate standard deviations across ten iterations. |
| 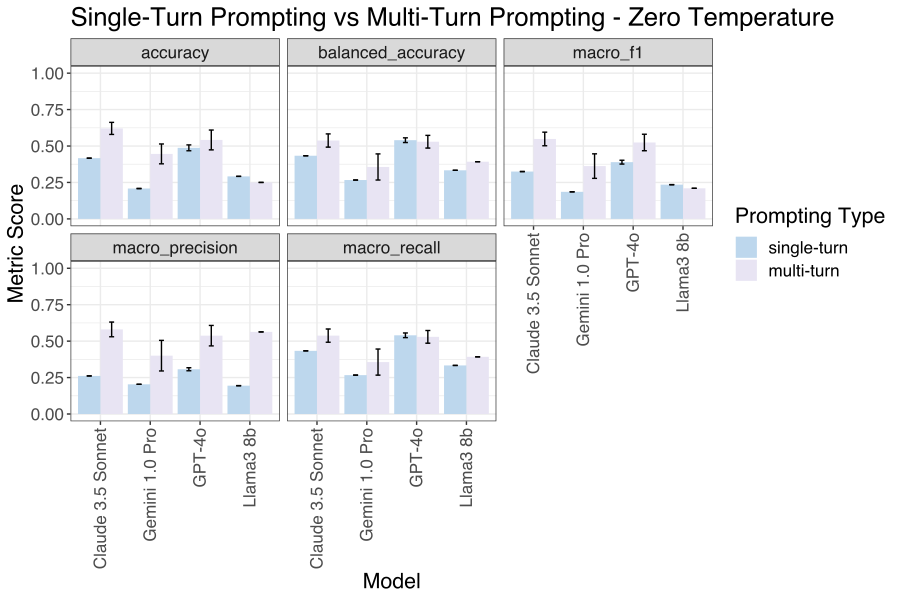 |
| **Supplementary Figure 4.** Performance comparison of single-turn prompting versus feedback multi-turn prompting at zero temperature and for self-regulated TF-target pairs. Accuracy, balanced accuracy, macro f1, macro precision, and macro recall are reported for the four LLMs (Claude, Gemini, GPT-4o, and Llama). As with performance at default temperature, multi-turn prompting results in higher accuracy for all models other than Llama. |
| 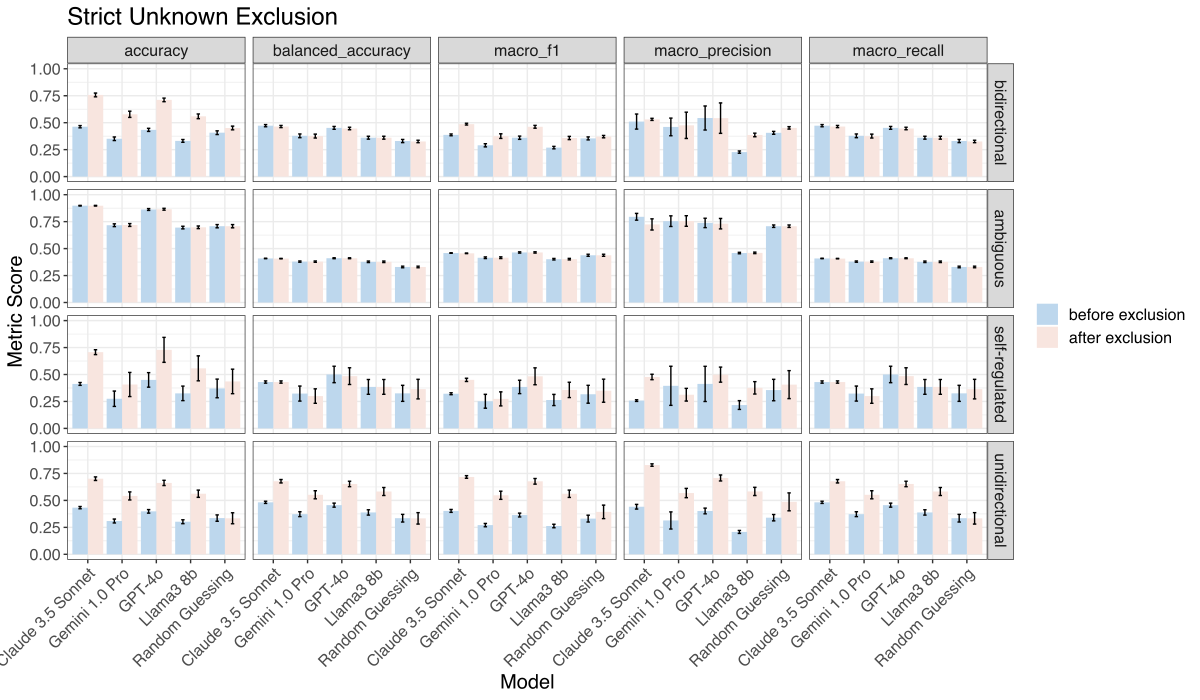 |
| **Supplementary Figure 5.** Performance and standard deviations for setting 2: Strict Unknown Exclusion, at default temperature. Performance is evaluated using five metrics, accuracy, balanced accuracy, macro F1, macro precision, and macro recall, across four regulatory categories: bidirectional, ambiguous, self-regulated, and unidirectional. Each panel compares model performance before (blue) and after (pink) excluding TF-target pairs under setting 2. Models include Claude, Gemini, GPT-4o, and Llama, and a random guessing baseline. The standard deviations across ten iterations are also reported. |
| 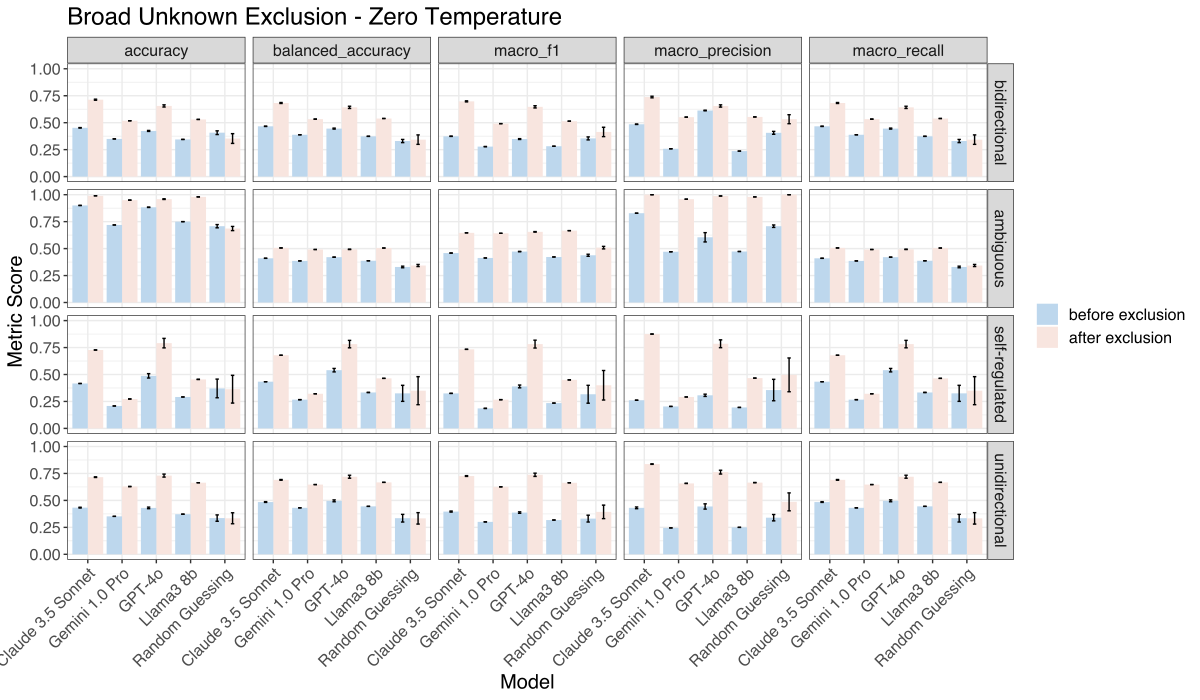 |
| **Supplementary Figure 6. Performance and standard deviations for setting 1: Broad Unknown Exclusion, at zero temperature.** Performance is evaluated using five metrics, accuracy, balanced accuracy, macro F1, macro precision, and macro recall, across four regulatory categories: bidirectional, ambiguous, self-regulated, and unidirectional. Each panel compares model performance before (blue) and after (pink) excluding TF-target pairs under setting 1. Models include Claude, Gemini, GPT-4o, and Llama, and a random guessing baseline. The standard deviations across ten iterations are also reported. |
| 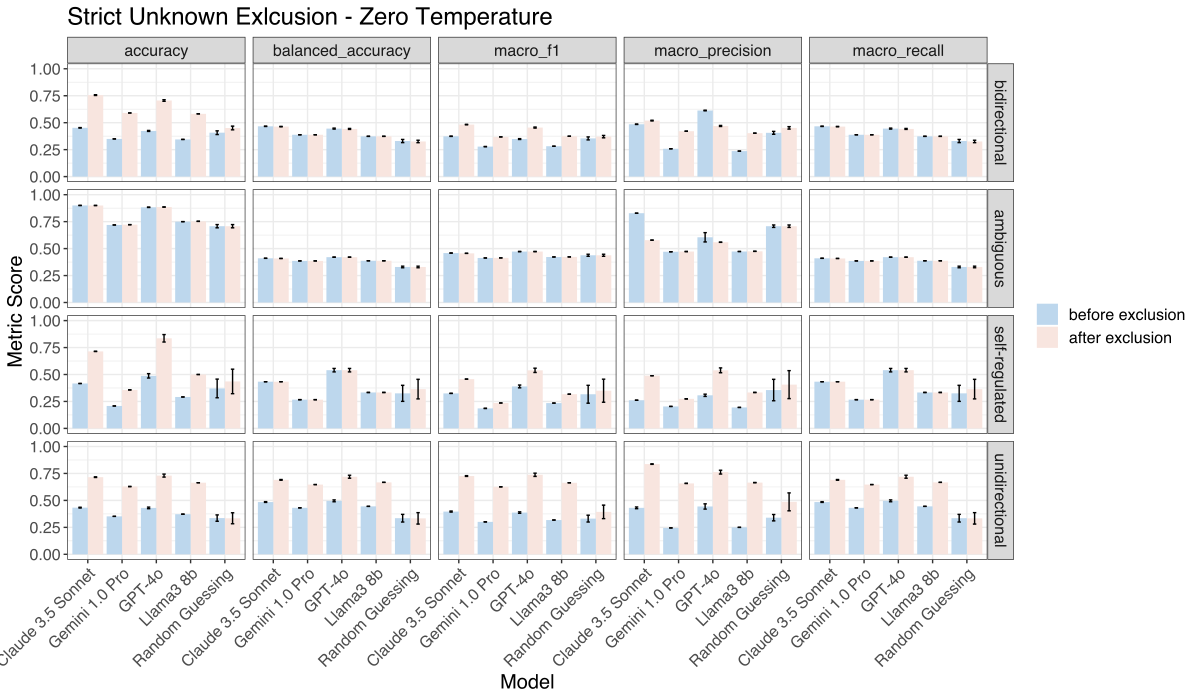 |
| **Supplementary Figure 7. Performance and standard deviations for setting 2: Strict Unknown Exclusion, at zero temperature**. Performance is evaluated using five metrics, accuracy, balanced accuracy, macro F1, macro precision, and macro recall, across four regulatory categories: bidirectional, ambiguous, self-regulated, and unidirectional. Each panel compares model performance before (blue) and after (pink) excluding TF-target pairs under setting 2. Models include Claude, Gemini, GPT-4o, and Llama, and a random guessing baseline. The standard deviations across ten iterations are also reported. |
| 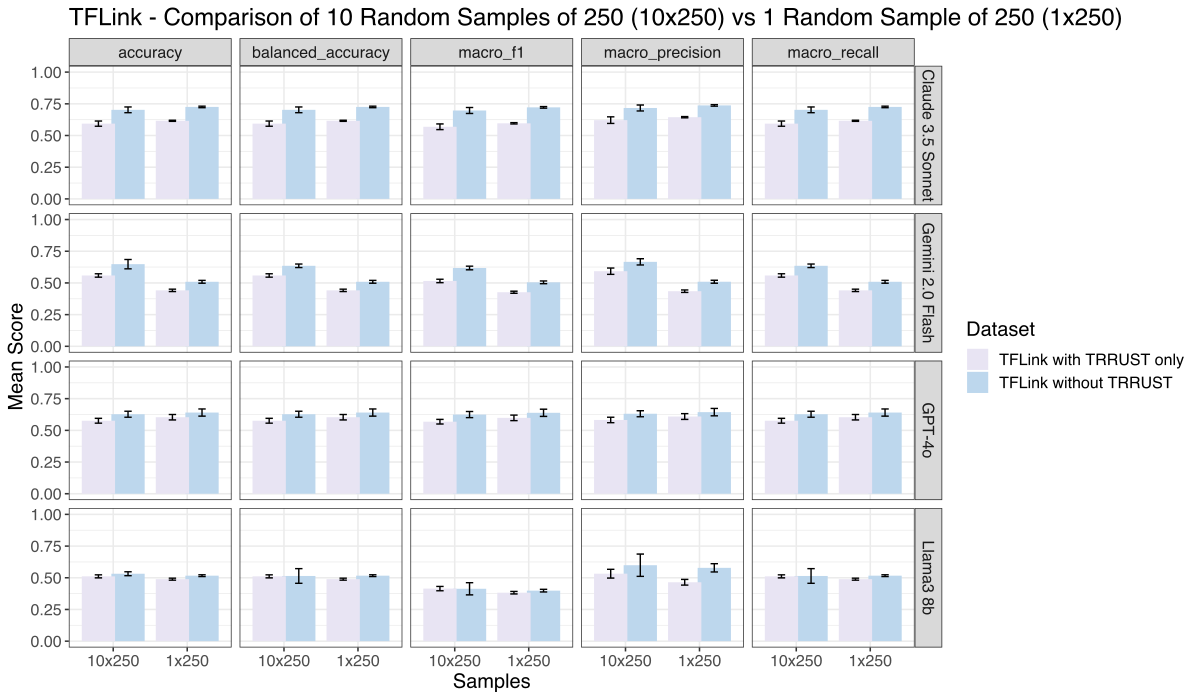 |
| **Supplementary Figure 8. Model performance across different sampling strategies on TFLink datasets.** Performance metrics (accuracy, balanced accuracy, macro F1, macro precision, and macro recall) are shown for four language models (Claude 3.5 SonnetClaude, Gemini 12.0 Flash, GPT-4o, and Llama3 8bLlama) under two datasets: TFLink with TRRUST only (purple) and TFLink without TRRUST (blue). Bars represent mean scores across ten iterations, and error bars indicate standard deviation. Results show that model performance is consistent across sampling strategies, suggesting that a single 250-pair sample is a reliable approximation of the larger dataset. |

| Supplementary Table 1. Llama3 8b performance on user prompts with and without the word “Please”, with standard deviations across ten iterations. | | | | | | |
| --- | --- | --- | --- | --- | --- | --- |
|  | With “Please” | Accuracy | Balanced Accuracy | Macro F1 | Macro Precision | Macro Recall |
| Bidirectional | Yes | 32.5 ± 2.0% | 36.0 ± 2.1% | 25.8 ± 1.8% | 22.6 ± 1.5% | 36.0 ± 2.1% |
|  | No | 33.1 ± 1.3% | 36.1 ± 1.3% | 26.9 ± 1.1% | 22.7 ± 1.1% | 36.1 ± 1.3% |
| Self-regulated | Yes | 29.2 ± 4.8% | 34.3 ± 5.2% | 23.9 ± 3.6% | 19.9 ± 2.8% | 34.3 ± 5.2% |
|  | No | 32.5 ± 6.7% | 38.5 ± 6.9% | 26.4 ± 5.2% | 21.7 ± 4.1% | 38.5 ± 6.9% |
| Unidirectional | Yes | 28.2 ± 0.7% | 37.6 ± 0.9% | 24.4 ± 0.6% | 30.2±13.7% | 37.6 ± 0.9% |
|  | No | 30.2 ± 1.8% | 38.8 ± 2.5% | 26.3 ± 1.6% | 20.8 ± 1.4% | 38.8 ± 2.5% |


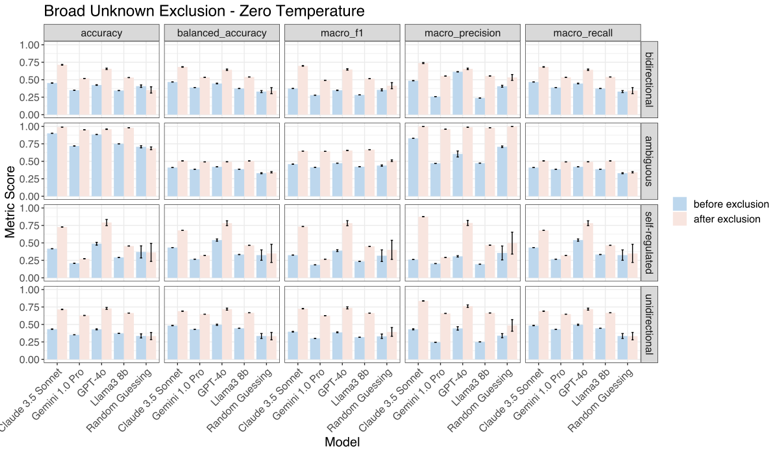


**Supplementary Figure 6. Performance and standard deviations for setting 1: Broad Unknown Exclusion, at zero temperature.** Performance is evaluated using five metrics, accuracy, balanced accuracy, macro F1, macro precision, and macro recall, across four regulatory categories: bidirectional, ambiguous, self-regulated, and unidirectional. Each panel compares model performance before (blue) and after (pink) excluding TF-target pairs under setting 1. Models include Claude, Gemini, GPT-4o, and Llama, and a random guessing baseline. The standard deviations across ten iterations are also reported.
